# Primary hyperoxaluria type I diagnosed after a kidney transplant presenting with subcutaneous calcification: a case report of sodium thiosulfate treatment

**DOI:** 10.3389/fphar.2025.1485024

**Published:** 2025-05-13

**Authors:** Min Wu, Jing Lu, Yu-Jia Wang, Yong-Qi Li, Qing Wei, Yu-Xiang Gong, Ri-Ning Tang

**Affiliations:** ^1^ Institute of Nephrology, Zhongda Hospital, School of Medicine, Southeast University, Nanjing, China; ^2^ Renal Pathology Department, Zhongda Hospital, School of Medicine, Southeast University, Nanjing, China; ^3^ Institute of Nephrology, Nanjing Drum Tower Hospital, Nanjing University Medical School, Nanjing, China

**Keywords:** primary hyperoxaluria, AGXT gene, subcutaneous calcification, skin biopsy, sodium thiosulfate

## Abstract

Primary hyperoxaluria (PH) is a rare autosomal recessive disorder that results from the overproduction of endogenous oxalate. The diagnosis of PH is often delayed or missed owing to its rarity, variable clinical expression and other diagnostic challenges. In this study, we report a patient with a frameshift variant, c.823_824dup, in the alanine-glyoxylate aminotransferase (AGXT) gene of PH1 who presented with renal failure recurrence after kidney transplantation, arteriovenous fistula (AVF) occlusion and subcutaneous calcification in adulthood. Skin biopsy revealed heavy deposition of calcium oxalate crystals in subcutaneous tissue without vascular oxalosis. After 6 courses of sodium thiosulfate (STS) treatment, X-rays of the bilateral hands showed the disappearance of subcutaneous calcification on the extremity of the left-hand ring-finger. This case highlights the importance of broad diagnostic testing prior to transplantation in patients who present with end-stage renal disease with unclear etiology. In addition, STS may be useful for PH1 patients with subcutaneous calcium deposits.

## 1 Introduction

Primary hyperoxaluria (PH) is a rare autosomal recessive disorder that results from the overproduction of endogenous oxalate due to genetic defects in enzymes critical for oxalate metabolism in the liver (Cochat and Rumsby, 2013). Subsequently, the storage of oxalate leads to recurrent kidney stones, nephrocalcinosis, kidney failure, and eventually life-threatening systemic disease (Cochat and Rumsby, 2013). The clinical presentation of PH is variable, ranging from infantile nephrocalcinosis to recurrent nephrolithiasis and progressive kidney disease in adulthood (Cochat and Rumsby, 2013). The diagnosis of PH is often delayed or missed owing to its rarity, variable clinical expression and other diagnostic challenges (Cai et al., 2019). Occasionally, the diagnosis is made after a patient progresses to end-stage renal disease (ESRD) or even after the disease recurs following kidney transplantation (Cai et al., 2019; Stone et al., 2021).

To date, three forms of PH have been characterized and named PH1, PH2, and PH3 (Cochat and Rumsby, 2013). PH1 is the most frequent and severe form, accounting for 70%–80% of all PH cases (Hoppe et al., 2009). PH1 is caused either by deficiency of the liver-specific peroxisomal alanine-glyoxylate aminotransferase (AGXT) or by its mistargeting from the peroxisome to the mitochondria (Groothoff et al., 2023). With disease progression, systemic oxalate deposition develops everywhere in the body of PH1 patients (Cochat and Rumsby, 2013). However, the early diagnosis of PH1 is still challenging.

Here, we describe a patient with a frameshift variant, c.823_824dup, in the AGXT gene of PH1 who presented with renal failure recurrence after kidney transplantation, arteriovenous fistula (AVF) occlusion and subcutaneous calcification in adulthood. Moreover, we share our experience with the potential of sodium thiosulfate for treating subcutaneous calcification complications in PH1 patients.

## 2 Case description

A 37-year-old man with ESRD and nephrolithiasis presented to the Emergency Department with sudden occlusion of the AVF after hemodialysis treatment. He also reported tenderness of the subcutaneous nodule on the left-hand ring finger, joint pain in the lower limbs, and tooth loss, all of which had developed over the past several months.

He was diagnosed with nephrolithiasis at age 31 and underwent double J-tube implantation. No information is available on his out-of-province management of nephrolithiasis. At the age of 34, he was diagnosed with ESRD and received a living-unrelated donor kidney transplantation with an unremarkable perioperative course. However, his creatine started to rise after the operation, and hemodialysis started. During the 3 years before this referral, he received maintenance hemodialysis (MHD) treatment thrice weekly via the AVF. He had an older sister and a son. There was no family history of kidney disease, hypertension, or kidney stones.

**FIGURE 1:**
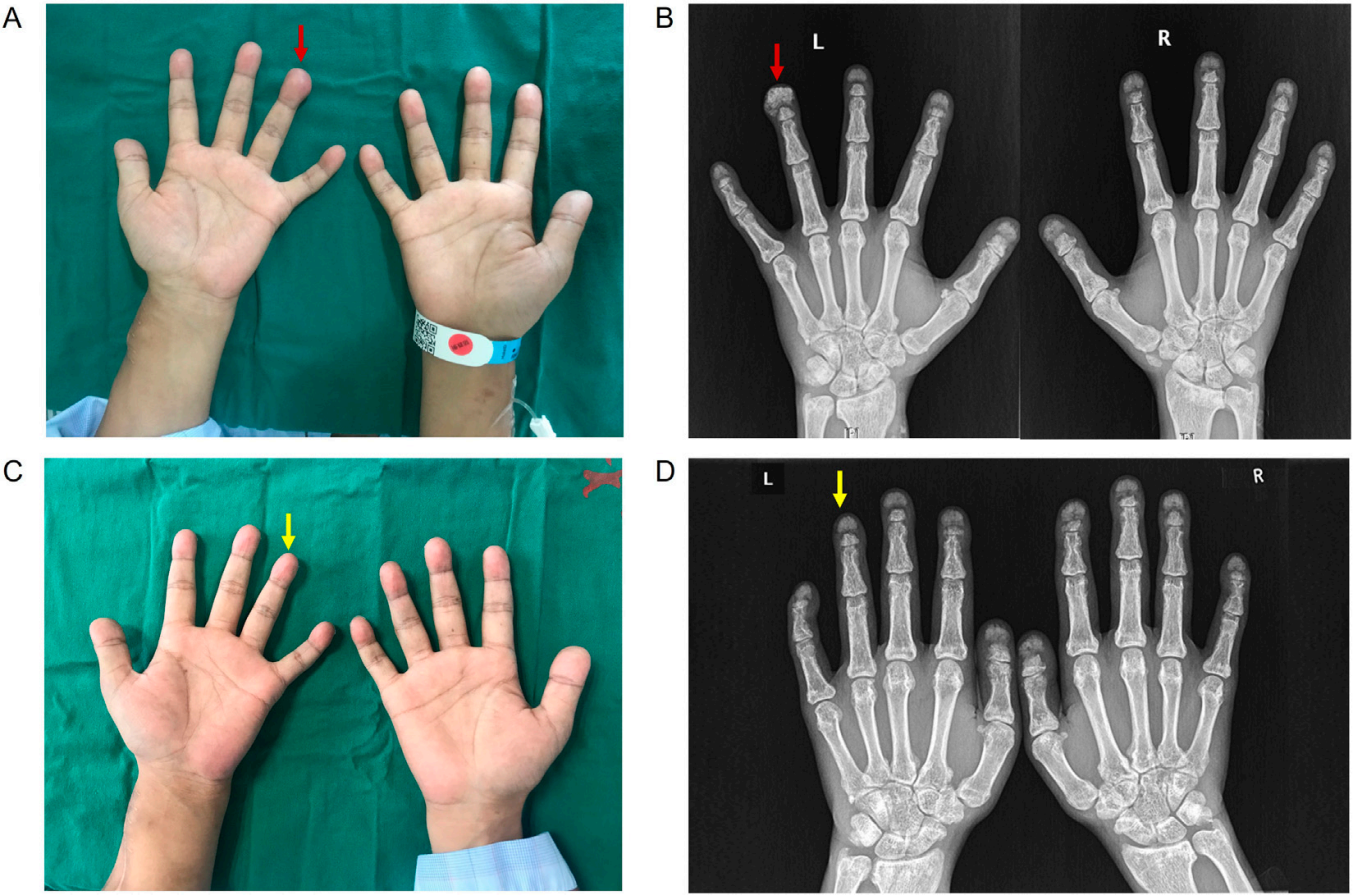
Clinical and imaging presentations of the patient. **(A)** The presence of subcutaneous nodule on left hand ring-finger. **(B)** X-rays of bilateral hands showed subcutaneous calcification on the extremity of left-hand ring-finger (red arrow). **(C)** Subcutaneous nodule on left hand ring-finger disappeared after STS treatment. **(D)** X-rays of bilateral hands showed the disappearance of subcutaneous calcification on the extremity of left-hand ring-finger after STS treatment (yellow arrow).

Physical examination revealed the absence of incisors, the presence of subcutaneous nodules on the left-hand ring-finger (Figure 1A), and tenderness of the knees and ankles. Otherwise, he presented with normal vital signs and cardiac, respiratory and abdominal examinations. There was no pulse of the AVF on the left forearm. Dorsalis pedis pulses were palpable, and the distal extremities were warm.

Biochemical analysis revealed a serum total protein concentration of 43.9 g/L. His serum albumin was 24.5 g/L, and his serum creatinine was 888 μmol/L. A fibrinolytic function test revealed a significantly increased D-dimer level of 12,029 μg/L. His serum bone alkaline phosphatase level was 33.99 μg/L. The following serologies were normal or negative: antinuclear antibody panel, rheumatoid factor, C3 and C4 complements, cryoglobulins, ANCA, anti-GBM, HIV antigen and antibody, hepatitis C, syphilis, and hepatitis B surface antigen. Serum protein electrophoresis showed no monoclonal gammopathy.

**FIGURE 2:**
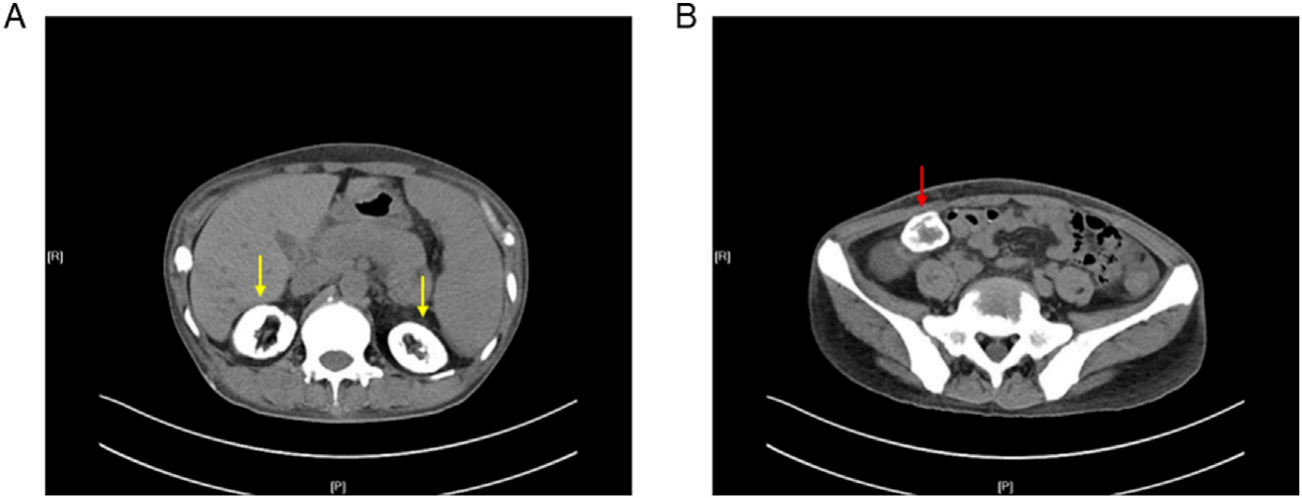
Computed tomography of the abdomen and pelvis showed nephrocalcinosis in **(A)** both kidneys (yellow arrow) and **(B)** transplanted kidney (red arrow).

X-rays of the bilateral hands showed subcutaneous extraosseous calcification on the extremity of the left-hand ring finger (Figure 1B). Computed tomography (CT) revealed nephrocalcinosis in both kidneys and transplanted kidney (Figure 2). No vascular calcification was observed. CT angiography revealed no stenosis or embolic source. A transthoracic echocardiogram revealed a normal left ventricular size and a normal systolic function.

**FIGURE 3:**
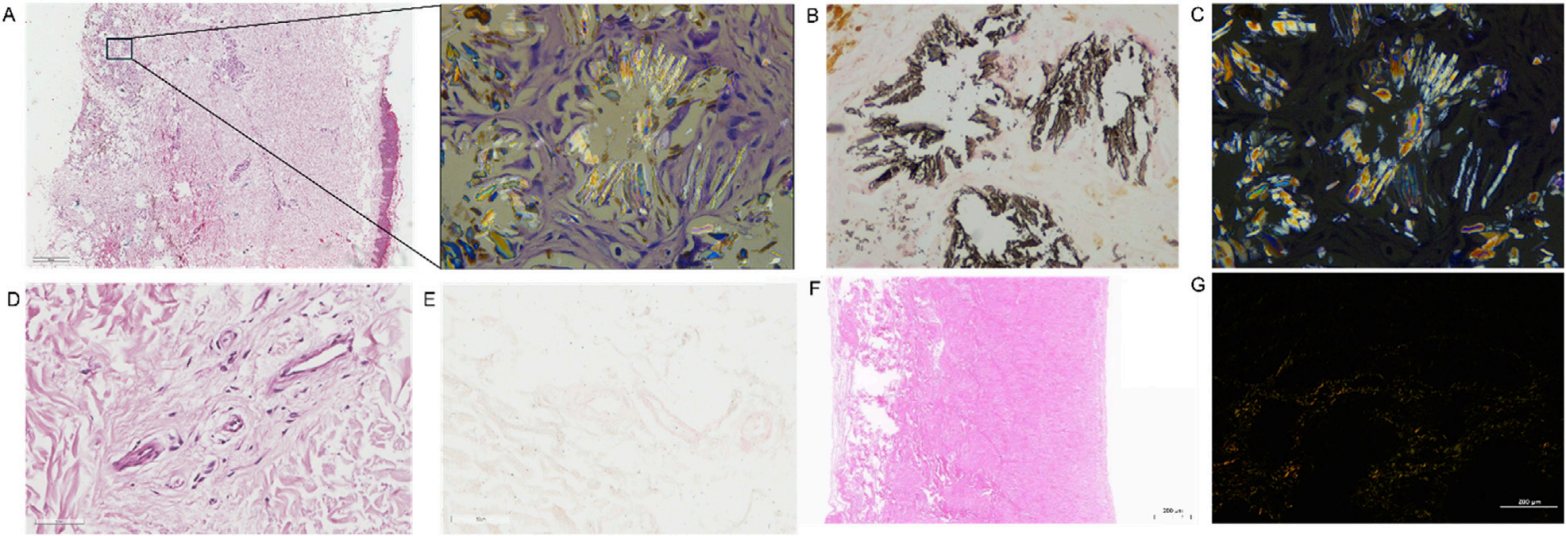
Examination of the calcium oxalate crystals on skin biopsy and AVF sample. **(A)** HE staining showed the deposition of crystal substance in skin biopsy sample. **(B)** Von Kossa staining showed black calcified deposits in skin biopsy sample. **(C)** The representative picture of birefringence on polarized microscopy in skin biopsy sample. The calcified crystals polarize and thus are calcium oxalate. **(D,E)** The representative image of HE **(D)** and Von kossa staining **(E)** in subcutaneous artery from skin biopsy sample. **(F,G)** The representative image of HE staining **(F)** and birefringence on polarized microscopy in AVF samples **(F,G)**.

Skin biopsy from the leg revealed heavy deposition of calcium oxalate crystals in subcutaneous tissue with positive von Kossa staining and birefringence on polarized microscopy (Figures 3A–C). Blood vessel samples were collected at the time of AVF reconstruction. Calcium oxalate deposition was not detected in vessels from skin (Figure 3D) or AVF samples (Figures 3E-G).

**FIGURE 4:**
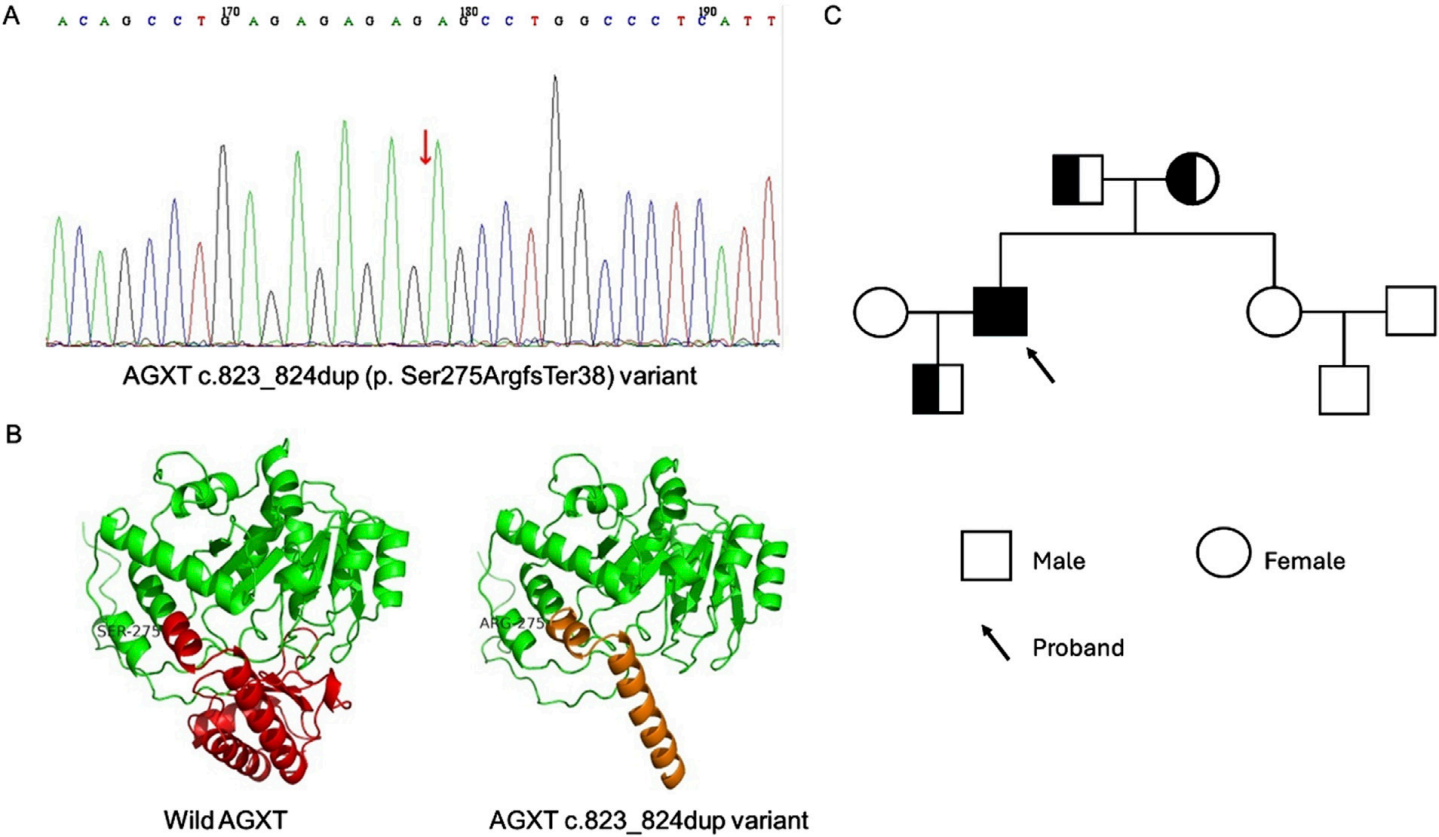
The Whole Exome Sequencing (WES) of the patient and family members and structural evaluation of AGXT variant. **(A)** WES revealed AGXT c.823_824dup (p. Ser275ArgfsTer38) variant in patient (red arrow). **(B)** Visualization of the wild and c.823_824dup variant of AGXT gene using swiss-model. **(C)** The pedigrees of the family with digenic inheritance.

Considering the unclear etiology of renal failure after kidney transplantation and unusual clinical presentation, whole-exome sequencing (WES) was performed. The results revealed a homozygous AGXT c.823_824dup (p. Ser275ArgfsTer38) variant (Figure 4A). A visualization of this frameshift variant is shown in Figure 4B. Sanger sequencing analysis of the family members revealed a heterozygous variant of AGXT, c.823_824dup, in the patient’s father, mother and son (Figure 4C).

After AVF reconstruction surgery, high-efficiency and high-flux HD treatment with low-molecular-weight heparin and an anticoagulant was started 4 times per week. Rivaroxaban was added to ameliorate the hypercoagulable state on interval days of HD. The herbal combination of achyranthes bidentata (Huai Niu Xi), angelica sinensis (Dang Gui), astragalus membranaceus (Huang Qi), and white paeony (Bai Shao) was administrated to ameliorate joint pain. In addition, this patient started to receive the sodium thiosulfate (STS) regimen following the “Zhongda STS approach” in May 2023. In detail, 6.4 g of STS dissolved in 100 mL of saline was administered intravenously once a day. One treatment course lasted 2 weeks, followed by a treatment-free period of 4 weeks. After 6 months of STS, there were no adverse effects, and the patient remained dialysis dependent. Bilateral X-rays revealed the disappearance of subcutaneous extraosseous calcification on the extremity of the left-hand ring finger (Figures 1C,D). A fibrinolytic function test revealed a decrease in the D-dimer level to 7,504 μg/L. His serum bone alkaline phosphatase level was 30.89 μg/L. The patient reported an ameliorated joint pain in his lower limbs after treatment. During a follow-up of 6 months, there was no subcutaneous nodule recurrence.

## 3 Discussion

Primary hyperoxaluria can present at any age with genotypic heterogeneity and variable phenotypes. Urolithiasis and nephrocalcinosis are the most common symptoms present in 90% and 48% of patients at diagnosis, respectively (Lieske et al., 2005). PH1 is reported to be the most severe type of PH, with a 65% prevalence in adults (Zhao et al., 2016). Late diagnosis in adults is usually due to inadequate metabolic screening in stone formers and deficient genetic testing in individuals without a family history of PH1. In the present case, the patient was diagnosed with PH1 through genetic testing after 3 years of chronic hemodialysis following the development of early kidney graft failure. Thus, broad diagnostic testing prior to transplantation is critical for patients who present with ESRD with unclear etiology.

PH1is caused by pathogenic variants in the AGXT gene, which impair the function of hepatic alanine-glyoxylate aminotransferase (AGT). Consequently, excessive glyoxylate is converted to oxalate. It is noteworthy that PH1 displays a heterogeneous phenotype. Currently, more than 300 pathogenic variants in the AGXT gene are described and most of them are missense mutations (Cellini, 2024). Previous OxalEurope studies observed a better prognosis of the p.Gly170Arg (G170R) missense mutation (Mandrile et al., 2014). Hopp et al. (2015) confirmed that the AGXT p.G170R mistargeting alleles resulted in a milder PH-1 phenotype; however, other putative AGXT mistargeting variants were associated with a more severe disease. According to recent findings of the European Hyperoxaluria Consortium, patients with c.731T>C (p.Ile244Thr) homozygote variants had better kidney survival than null homozygotes (Metry et al., 2023). Given the variable clinical expression of these genes, these findings suggest a possible genotype‒phenotype association in PH1 patients. In the present case, PH1 patients with the AGXT c.823_824dup (p. Ser275ArgfsTer38) variant exhibited nephrocalcinosis, ESRD, a hypercoagulant state, metabolic bone disorder and subcutaneous nodules with ectopic calcification. Xin and colleagues reported a PH1 patient diagnosed at the age of 0.5 with the same c.823_824dup variant presenting bilateral stones, urinary tract infection, hematuria, hydronephrosis and lived with stable disease (Xin et al., 2023). Thus, the ultimate phenotype and prognosis of patients may also be affected by the age of diagnosis and clinical intervention.

Cutaneous and subcutaneous manifestations are rare in PH1, with only a few case reports (Poyah et al., 2021; El-Saygeh et al., 2021; Baethge et al., 1988; Spiers et al., 1990; Somach et al., 1995; Farrell et al., 1997; Marconi et al., 2002; Bogle et al., 2003; Rubenstein et al., 2004; Blackmon et al., 2011). Most patients present with livedo reticularis, acrocyanosis, and extremity pain caused by vascular oxalosis. However, in our patient, the skin biopsy showed heavy deposition of calcium oxalate crystals in the subcutaneous tissue without vascular oxalosis. It is well-known that kidney, bone, eyes and myocardium are the main targets for oxalate storage in patients with PH. But oxalate crystals have also been found in skin (Mandrile et al., 2023). Taken together, these evidence supported the value of skin biopsy in determining the pathological diagnosis of cutaneous and subcutaneous manifestations in patients with PH1. But the invasive property of this procedure limits the performance of re-biopsy to observe the efficacy of therapy. Further studies are required to determine the non-invasive biomarkers for the evaluation of calcium oxalate crystal deposition in subcutaneous tissue in patients with PH.

STS, a potent antioxidant and chelator of calcium, has been used to prevent the progression of calcified lesions in calcific uremic arteriolopathy (CUA) (Pasch et al., 2008; Hayden et al., 2005). Although randomized controlled trials have not been performed, the benefits of STS for patients with CUA or ectopic calcification have been highlighted in case reports and observational studies (Nigwekar et al., 2013; Zitt et al., 2013). Considering the pathological role of subcutaneous calcium oxalate deposition in this patient, STS was administered at a dosage of 6.4 g once a day following the “Zhongda approach” for CUA treatment in our departmen (Yang et al., 2022). After 6 courses of STS treatment, X-rays of the bilateral hands showed the disappearance of subcutaneous calcification on the extremity of the left-hand ring finger in this patient. To the best of our knowledge, this is the first report on the efficacy of STS for subcutaneous calcification in PH1 patients. This evidence suggests the potential use of STS for treating calcified complications in PH1 patients.

The mechanisms underlying this protective role of STS in ameliorating calcified lesions are not fully understood. Previously, STS has been demonstrated to chelate calcium deposits into soluble calcium thiosulphate complexes and enhance urinary calcium loss (Pasch et al., 2008; Vaz et al., 2025). Moreover, STS exhibited an antioxidant efficacy by scavenging ROS and producing glutathione to attenuate ectopic calcification (Hayden et al., 2005). Recently, Liu et al. (2023) showed that STS alleviated the progression of vascular calcification in hemodialysis patients by the modulation of calcification factors expression. In addition, STS could induce the generation of hydrogen sulfide with vasodilatory, anti-inflammatory and analgesic properties (Song et al., 2023).

In conclusion, we present this case as a reminder of PH1 as a rare disorder and its atypical presentations. As genetic testing for PH is now widely available at a relatively low cost and has a short turnaround time, we propose that genetic screening for PH1 should be considered for any transplant candidate with ESRD of uncertain etiology. In addition, STS may potentially be useful for PH1 patients with subcutaneous calcium deposits.

## Data Availability

The original image and data are included in the article, further inquiries can be directed to the corresponding author.
